# Classification and clinical features of headache patients: an outpatient clinic study from China

**DOI:** 10.1007/s10194-011-0360-2

**Published:** 2011-07-09

**Authors:** Yunfeng Wang, Jiying Zhou, Xiaoping Fan, Xuelian Li, Li Ran, Ge Tan, Lixue Chen, Kuiyun Wang, Bowen Liu

**Affiliations:** Department of Neurology, The First Affiliated Hospital of Chongqing Medical University, Chongqing, 400016 China

**Keywords:** Outpatient, Headache, Cross-sectional study, Clinical feature, Migraine

## Abstract

This study aimed to analyze and classify the clinical features of headache in neurological outpatients. A cross-sectional study was conducted consecutively from March to May 2010 for headache among general neurological outpatients attending the First Affiliated Hospital of Chongqing Medical University. Personal interviews were carried out and a questionnaire was used to collect medical records. Diagnosis of headache was according to the International classification of headache disorders, 2nd edition (ICHD-II). Headache patients accounted for 19.5% of the general neurology clinic outpatients. A total of 843 (50.1%) patients were defined as having primary headache, 454 (27%) secondary headache, and 386 (23%) headache not otherwise specified (headache NOS). For primary headache, 401 (23.8%) had migraine, 399 (23.7%) tension-type headache (TTH), 8 (0.5%) cluster headache and 35 (2.1%) other headache types. Overall, migraine patients suffered (1) more severe headache intensity, (2) longer than 6 years of headache history and (3) more common analgesic medications use than TTH ones (*p* < 0.001).TTH patients had more frequent episodes of headaches than migraine patients, and typically headache frequency exceeded 15 days/month (*p* < 0.001); 22.8% of primary headache patients were defined as chronic daily headache. Almost 20% of outpatient visits to the general neurology department were of headache patients, predominantly primary headache of migraine and TTH. In outpatient headaches, more attention should be given to headache intensity and duration of headache history for migraine patients, while more attention to headache frequency should be given for the TTH ones.

## Introduction

Headache is experienced by the majority of the population and has a major impact on public health. Worldwide, the condition has been ranked among the ten most disabling conditions by the World Health organization (WHO). In the global population, the prevalence of active headache disorder is 47%, migraine 10%, tension-type headache (TTH) 38% and chronic headache 3%; the disability attributable to TTH is larger worldwide than that due to migraine [1]. Furthermore, headache is the most common neurological symptom presented by patients to general practitioners and neurologists [2].

Although headache disorders affect the majority of the adult population, most headaches can be self-managed and do not require medical intervention. Patients who consult doctors have different clinical features and the distribution of headache types seen in the clinic differs from population-based studies. A study by an ED in Greece [3] reported that TTH was the most frequent diagnosis, followed by secondary headache and migraine. In a clinical study from a headache center in east Hungary [4], migraine was the most common headache, while TTH patients reported more severe disability than migraines. A study from Asia [5] also showed that migraine was the most common headache diagnosis in neurological services. A population survey in Chile [6] indicated that migraineurs were more likely to consult doctors than TTH patients. A study from Denmark [7] reported that 56% of migraine patients and 16% of patients with TTH were seen by their family practitioner, whereas 16% of migraine and 4% of TTH patients sought specialist consultation.

Nevertheless, little is known about the distribution of different types of headache, including primary and secondary headaches, in outpatient departments where most patients in China first present. The aim of this survey was therefore to record the demographic data of patients attending for consultation of headache treatment in the general neurological outpatient department of a tertiary care hospital. Because comparison of the clinical characteristics of the different headache types could aid the development of headache treatment guidelines for public health intervention, the study also analyzed the clinical features of patients with migraine and TTH.

## Patients and methods

A cross-sectional study was conducted consecutively from March to May 2010 for headache among patients attending the neurological outpatient department at the First Affiliated Hospital of Chongqing Medical University. Each participant was interviewed by a qualified headache doctor and completed a self-administered questionnaire. Diagnosis was performed by doctors participating in the patient interviews and the diagnosis of CDH was confirmed by a headache specialist (corresponding author). The questionnaire was in three sections. The first included demographic details and past health status. The second comprised a headache profile, including duration of headache history (<1, 1–3, 4–6, >6 years), attack frequency (average number of headache days per month: <1, 2–4, 5–14, or ≥15 days/month) and severity using a 0–10 visual analog scale (VAS); pulsating, pressing/tightening or other headaches; presence or absence of headache aggravation from climbing stairs or routine physical activities; location (unilateral, bilateral or orbital), duration of headache attacks (<30min, 30 min to 2 h, 2–4 h, 4–72 h or >3 days) and accompanying symptoms (presence or absence of nausea, vomiting, photophobia or phonophobia, and clinical signs of cranial sympathetic dysfunction such as conjunctival injection, lacrimation, nasal congestion, rhinorrhoea, forehead and facial sweating, miosis, ptosis, eyelid edema). In addition, four major migraine aura symptoms (visual, sensory, motor and speech symptoms) were also queried. The third section concerned analgesic medication usage (never used, <1, 1, 2, ≥3 days/week), sleep status (excellent, good, fair, poor) and family history of headache.

ICHD-II [8] was applied to divide the patients into subgroups. Primary headache included migraine, TTH, cluster headache and other primary headaches [including primary stabbing headache, cough headache, hypnic headache, new daily persistent headache (NDPH) and others]. Secondary headache was diagnosed if the patient’s headache could be attributed to viral infection, cranial neuralgias, cranial or cervical vascular disorder, psychiatric disorder, sinusitis or alcohol ingestion. If the headache could not be accurately categorized as either primary or secondary, it was classified as headache not otherwise specified (headache NOS).

Patients who reported a headache frequency of at least 15 days/month over a period lasting more than 3 months were classified as having chronic daily headache (CDH). CDH included chronic migraine (CM), chronic tension-type headache (CTTH), medication-overuse headache (MOH), chronic cluster headache and NDPH. CM was defined by the following criteria revised by Olesen [9]: (1) headache (tension type and/or migraine) on ≥15 days/month for at least 3 months; (2) migraine that fulfilled ICDH-II and was experienced on ≥8 days/month for at least 3 months; (3) no medication overuse.

For analysis, we defined the following two rules: (1) patients who fulfilled migraine diagnostic criteria, but also expressed other headache characteristics such as TTH, were categorized as having migraine; (2) probable migraine was also included with migraine. The study protocol was approved by the Ethical Committee at Chongqing Medical University and complied with the Declaration of Helsinki. All patients gave their informed consent for this study.

Statistical analysis of data was performed using the SPSS17.0 statistics package for PC. Demographic data were summarized using descriptive statistics. Quantitative data were presented as mean ± SD. The Student’s *t* test and the χ^2^ test were used for comparing quantitative and qualitative data, respectively. Separate variance estimation (*t*’ test) was used to detect possible differences in mean ages between primary headache and headache NOS, which had unequal variance. Mann–Whitney *U* tests were performed for ordinal categorical variables to identify the clinical features differing between migraine and TTH. All calculated *p* values were two tailed and statistical significance was defined as a *p* value of <0.05.

## Results

### Demographic characteristics of headache patients

Over the period of our study, 9,282 patients presented to the neurological outpatient department. Of these, 1,806 (19.5%) reported headache as the primary reason for their visit. All these patients were interviewed; the questionnaire response rate was 93.19% (1,683/1,806). A total of 1,683 cases of headache patients were investigated; 640 (38%) patients were seeking medical advice regarding their headache for the first time, 960 (57.1%) patients had consulted for their headache previously and 83 (4.9%) patients were unable to recall their headache consultation history. CT scan or MRI imaging to detect or exclude brain disorders was performed in 99 (10.3%) of the 960 patients who had attended previously.

The age and gender distributions of the different headaches types are shown in Table 1. Primary headache patients accounted for 50.1% of all headache patients in our study, and migraine and TTH accounted for 23.8% (401/1,683) and 23.7% (399/1,683) of the total, respectively. Secondary headache (27%) was the next most frequently diagnosed headache type, and headache NOS accounted for 22.9%.

**Table 1:** The gender and age distribution of headaches

Diagnosis	Age (mean ± SD)	Gender		
		Male N (%)	Female *N* (%)	Both *N* (%)
Primary headache				
Migraine	42.17 ± 13.14*	78 (19.5)	323 (80.5)	401 (23.8)
TTH	44.78 ± 12.78	132 (33.1)	267 (66.9)	399 (23.7)
Cluster headache	36.13 ± 9.6	7 (87.5)	1 (12.5)	8 (0.5)
Other headache	43.91 ± 15.48	14 (40.0)	21 (60.0)	35 (2.1)
Secondary headache	51.39 ± 15.15	146 (32.2)	308 (67.8)	454 (27.0)
Headache NOS	46.02 ± 15.45	143 (37.0)	243 (63.0)	386 (22.9)
Total	46.17 ± 14.62	520 (30.9)	1,163 (69.1)	1,683 (100)

* Age difference between migraine and TTH, *p* < 0.05

**Fig. 1:**
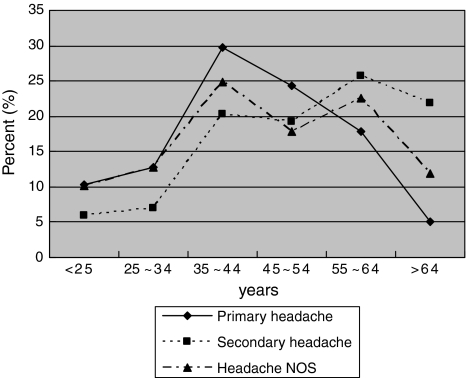
Age distribution of headache patients

Female patients (69.1%, 1,163/1,683) predominated across all headache patients; there was no age difference between genders (males 45.95 ± 15.69 years, females 46.26 ± 14.12 years). Primary headache patients were generally younger than secondary headache and headache NOS patients (43.42 ± 13.11 vs. 51.39 ± 15.15 years, *p* < 0.001; 43.42 ± 13.11 vs. 46.02 ± 15.45 years, *p* = 0.004 by *t*’ test). There was a clear pattern in the age distribution, with the number of primary headaches peaking in the age range 35–44 years. By contrast, the highest numbers of patients attending for secondary headache were in the age range 55–64 years (Fig. 1).

### Clinical features of migraine and TTH

Of the patients of migraine, 215 (53.6%) had migraine without aura; 163 (40.7%) of the patients with probable migraine 23 (5.7%) had migraine with aura and 58 (14.5%) patients had migraine coexisting with TTH.

Of the study group, 68.6% (267/389) of migraine patients and 64.9% (253/390) TTH patients had previously consulted for headaches (*p* > 0.05); 2.6% (21/800) migraine and TTH patients were unable to recall their headache consultation history. Only 1.4% (11/800) had used preventive drugs, and this was flunarizine in all cases; none had used triptans for acute pain relief.

**Fig. 2:**
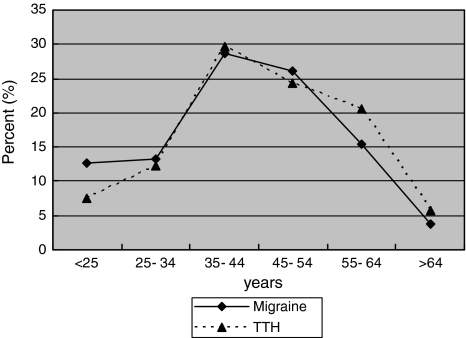
Age distribution of migraine and TTH

Although both migraine and TTH patients were predominantly in the age range of 35–44 years (Fig. 2), migraine patients were somewhat younger than TTH patients (42.17 ± 13.14 vs. 44.78 ± 12.78 years, *p* < 0.05). Compared to TTH, there was a significantly higher proportion of females among the migraine patients (80.5 vs. 66.5%, *p* < 0.001).

Table 2 presents the different clinical features of migraine and TTH headaches. Compared to TTH patients, migraine patients reported a longer duration of headache history, were more likely to use analgesic medication and experienced headache attacks of greater intensity. Further analysis showed that more migraine patients than TTH patients had a headache history of ≥6 years (50.6 vs. 35.3%, *p* < 0.001), whereas the proportion of patients with a headache frequency ≥15 days/month was higher in TTH patients than in the migraine group (54 vs. 35%, *p* < 0.001).

**Table 2:** Different clinical features between migraine and TTH

	Migraine (*N*)	%	TTH (*N*)	%	*p* values
Duration of headache history (years)					
<1	55	13.7	70	17.5	<0.001*
1–3	79	19.7	126	31.6	
4–6	64	16.0	62	15.5	
>6	203	50.6	141	35.3	
Intensity	6.29 ± 1.34		4.22 ± 1.52		<0.001
Frequency (days/month)					
≤1	100	26.10	50	13.00	<0.001*
2–4	63	16.40	49	12.70	
5–14	86	22.50	78	20.30	
≥15	134	35.00	208	54.00	
Analgesic medication uses (days/week)					
No use	173	43.1	234	58.6	<0.001*
<1	131	32.7	81	20.3	
1	37	9.2	21	5.3	
2	13	3.2	10	2.5	
≥3	47	11.7	53	13.3	
Family history					
No	235	59.3	295	75.4	<0.001
Yes	161	40.7	96	24.6	

* Mann–Whitney *U* test

### Chronic daily headache

Of the primary headache patients, 22.8% (192/843) were given an additional diagnosis of CDH by a headache specialist. As shown in Table 3, CTTH (64.1%, 123/192) was the most common CDH, followed by MOH (23.4%, 45/192). Females also outnumbered males among the CDH patients (70.3 vs. 29.7%, *p* < 0.001), a gender ratio similar to that seen with episodic headaches. The mean age of CDH was higher than patients with episodic headache (46.75 ± 12.27 vs. 42.45 ± 13.26 years, *p* < 0.001). The mean age of NDPH patients was the youngest, while MOH patients had a higher mean age than either NDPHs or CTTHs (Table 3).

**Table 3:** Age and gender distribution of chronic daily headache (CDH)

CDH	Total *N* (%)	Male *N* (%)	Female *N* (%)	Age (mean ± SD)
CM	9 (4.7)	3 (33.3)	6 (66.5)	48.89 ± 7.72
CTTH	123 (64.1)	35 (28.5)	88 (71.5)	46.38 ± 12.19
MOH	45 (23.4)	12 (26.7)	33 (73.3)	51.00 ± 10.66*
NDPH	15 (7.8)	7 (46.7)	8 (57.3)	35.73 ± 13.23**
Total	192 (100)	57 (29.7)	135 (70.3)	46.75 ± 12.27

* MOH patients were older than CTTHs and NDPHs (*p* < 0.05, ANVOA analysis)

** NDPH patients were younger than other CDHs (*p* < 0.01, ANVOA analysis)

Of the MOH patients, 20% (9/45) had primary headache of migraine, 75.6% (34/45) TTH, and 4.4% (2/45) NDPH. With regard to the acute treatment drug types, 91.1% of MOH patients used combination analgesic medications, 8.9% used non-steroid anti-inflammatory drugs (NSAIDs) and none used triptans or ergot.

## Discussion

In our survey, 19.5% patients gave headache as their primary reason for visiting the outpatient department. This is similar to the proportion of headache patients, 12.7 and 15.5%, respectively, among patients to EDs in Italy [10] and Greece [3]. Because the health-care system in China permits patients to visit a medical center without referral from basic medical units, all patients were self-referred. Our outpatient study is therefore comparable to studies of patients attending EDs. However, a study in Japan which has a similar health-care system reported that only 8.9% of neurological outpatients gave headache as their primary reason for attending [11]. Our result showed that headache should be considered as one of the most important clinical issues for neurological outpatient physicians in china.

The percentage of secondary headache (27%) in this study may be considered high. However, results of some other reports are similar to ours. Studies on ED patients found that 22.1% [3], 25% [12] and 41.3% [10] of patients were attending for secondary headache. An outpatient headache clinic [13] and a general practice [11] reported 9.4 and 42%, respectively (Table 4). We surmise that both the outpatient physician in our study and ED physicians are trained to recognize headache secondary to acute neurological disease first, whereas the diagnosis and subclassification of primary headache are primarily performed by headache clinics. It can often be difficult to distinguish between primary and secondary headaches, particularly in cases of acute headache and in patients aged more than 50 years. One study has suggested that headache patients older than 50 years have a fourfold higher risk that their headache is secondary to another predisposing condition [14]. Our results confirm that secondary headaches are more common than primary headaches in older patients.

**Table 4:** Headache diagnoses from different health-care departments (some data from Dermitzakis [3])

Diagnosis	ED, Greek, 2010 (%) [3]	ED, USA, 2005 (%) [12]	ED, Italy, 2004 (%) [10]	Headache clinic, USA 2011 (%) [13]	General medicine, Japan 2010 (%) [11]	Neurology outpatient, China 2010 (%)
Primary headache	50.1	64	24.3	73.4	39.9	50.1
Migraine	15.4	38.7	16.7	51.4	30.8	23.8
TTH	33.6	7.1		16	39.9	23.7
Secondary headache	22.1	25	41.3	9.4	42	27
Headache NOS	27.7	20	34.4	3.4	17.9	22.9

Headache NOS (22.9%) was also commonly encountered in our study. It is possible that this condition is over-represented because our survey was unable to provide a sufficiently detailed medical history and, upon further intensive investigation, only a proportion of these patients would be classified as headache NOS.

For primary headaches, migraine (23.8%) and TTH (23.7%) were the two most common headache types. Most previous studies have indicated that migraine was the most common headache type [4, 5, 13]. In previous reports from Asia, migraineurs represented the largest proportion of patients consulting their neurologists with a headache complaint (50.9–85.8%) [5, 15]. It is possible that migraine is preferentially diagnosed by specialist of headache centers. However, an ED study in Greece [3] reported that TTH was the most frequent diagnosis for primary headache. Bendtsen [16] considered TTH to be the most common but also the most neglected type of headache. Our findings argue that TTH and migraine should be given equal attention in outpatient departments and EDs.

With regard to the age distribution of migraine and TTH, the present study showed that primary headache was most commonly encountered in patients aged 35–44 years. This contrasts with an epidemiological study of migraine in Taiwan [17] where the highest prevalence of primary headache was in the age range 30–34 years; in another study the majority of TTH patients were aged 30–39 years [1]. Overall, however, these findings demonstrate that migraine and TTH patients seeking medical attention for headache tend to be older than the average age of community patients.

For all types of headache, with the exception of cluster headaches, females were over-represented. For migraine, the female:male ratio was 4:1, a value consistent with reports that 79% of patients visiting an outpatient health-care clinic [18] and 84% of patients attending a headache clinic [4] were female. For TTH patients in our study the female: male ratio was 2:1, whereas the general population data showed the ratio was 5:4 [19]. Overall, our data suggest that women are only marginally over-represented among TTH patients, whereas there was a significant gender bias in favor of females in patients with migraine.

### Different clinical features of migraine and TTH

Although TTH and migraine can exhibit many similar features [20], respond effectively to similar medications and could possibly share similar genetic predisposing factors, there were clear differences between the TTH and migraine patients. Previous population-based studies reported that the average headache frequency was <1 day/month in both migraine [17] and TTH [21]. However, in the present study, the majority of both migraine and TTH patients had headaches in excess of 1 day/month. Furthermore, the proportion of ≥15 day/month headache in TTH patients was higher than in migraineurs. We also find that migraine patients attending the outpatient department have a longer duration of headache history than TTH patients, and also have both increased use of analgesic medications and a family history of headache. Our results support the contention from a general population study [22] that migraine and TTH are distinct entities, and that migraine is an all-or-none phenomenon triggered with an individually variable threshold, whereas the severity of TTH increases according to headache frequency. Our results suggest that headache intensity and duration of headache history are the most relevant factors for migraine patients, whereas headache frequency is the most important index of severity in TTH patients.

### Chronic daily headache

Although 35% of migraine patients and 54% of TTH patients reported a headache frequency of ≥15 days/month, only 22.8% (192/1,683) of participants were defined as having CDH. This reflects our criteria for CDH that require the patient not only to have a headache frequency of ≥15 days/month, but also that the condition must have persisted for at least 3 months. CTTH was the most common condition, followed by MOH. Only 4.7% were defined to have CM. Similar clinical results were reported in Pakistan [15], while a study from India [23] found that CM was more common than CTTH. Using revised criteria for CM, the Danish headache center diagnosed only 3% of patients with CM [24], arguing that the revised criteria were significantly more restrictive. MOH was common in patients with chronic headache, and they were generally older than non-MOH patients. The most common type of medication overuse was associated with the use of combination analgesic medications, which are easy to acquire and have a high risk of overuse, as previously reported in a study on Chinese people from Taiwan [25].

### Limitations

The present study has several methodological limitations. First, because this is a hospital-based survey, the data cannot be used to estimate the prevalence of primary headaches in the general population. Second, the restrictive criteria for CDH employed in this study could underestimate the prevalence of this condition, most particularly of chronic migraine. Third, we did not evaluate the impact of the patient’s condition on their work, household and social activities, and such factors should be taken into consideration when evaluating the wider impact of headache. Fourth, we compared only a limited number of clinical features in migraine and TTH; further studies will be required to differentiate between migraine and TTH and the specific factors that lead the patients to seek medical attention.

## Abbreviations

CDH: Chronic daily headache
CM: Chronic migraine
CTTH: Chronic tension-type headache
MOH: Medication-overuse headache
TTH: Tension-type headache
NDPH: New daily persistent headache
ED: Emergency department
Headache NOS: Headache not otherwise specified
ICHD-II: International classification of headache disorders, 2nd edition
